# Pharmacological Treatment of Cerebellar Ataxia in Pediatric Ataxia‐Telangiectasia: A Systematic Review

**DOI:** 10.1111/ene.70683

**Published:** 2026-07-01

**Authors:** Fabiola Panvino, Roberto Paparella, Giorgia Urciuolo, Serena Galosi, Carlotta Spagnoli, Vincenzo Leuzzi, Francesco Pisani

**Affiliations:** ^1^ Department of Human Neuroscience Sapienza University of Rome Rome Italy; ^2^ Department of Maternal Infantile and Urological Sciences Sapienza University of Rome Rome Italy

**Keywords:** ataxia‐telangiectasia, cerebellar ataxia, pediatric, pharmacological treatment, systematic review

## Abstract

**Introduction:**

Ataxia‐telangiectasia (AT) is characterized by progressive cerebellar ataxia, oculomotor apraxia, immunodeficiency, and increased cancer susceptibility. No disease‐modifying treatment is available. This systematic review aimed to evaluate the efficacy and safety of pharmacological interventions for ataxia in pediatric AT.

**Method:**

A systematic search was conducted across MEDLINE, Scopus, Web of Science, and ClinicalTrials.gov through December 2025. Eligible studies included randomized controlled trials (RCTs) and single‐arm interventional studies evaluating pharmacological treatments for cerebellar ataxia in patients aged ≤ 18 years with genetically confirmed AT.

**Results:**

Thirteen studies (3 RCTs, 10 single‐arm trials) involving 314 participants (mean age 10.87 years) were included. Interventions included betamethasone, erythrocyte‐encapsulated dexamethasone (eDSP), nicotinamide riboside (NR), leucine derivatives, and amantadine. Betamethasone demonstrated transient improvements in the Scale for the Assessment and Rating of Ataxia (SARA) and International Cooperative Ataxia Rating Scale (ICARS) scores, with dose‐dependent systemic toxicity. eDSP showed favorable tolerability but was ineffective in phase 3 trials, although subgroup analyses suggested a potential benefit in children aged 6–9 years. NR supplementation improved SARA and AT Neurological Examination Scale Toolkit (AT‐NEST) scores in open‐label studies. Leucine derivatives showed mixed results. Amantadine showed benefits in patients with extrapyramidal symptoms.

**Conclusions:**

The recurrent pattern of promising open‐label findings followed by negative phase 3 results highlights a translational gap attributable to methodological limitations, including small sample sizes, heterogeneous populations, and variable outcome measures. The non‐linear disease progression and age‐dependent treatment response further complicate trial design. Current evidence remains insufficient to guide clinical practice.

**Trial Registration:**

PROSPERO number: CRD420251184721

**Abbreviations**
ADLL: acetyl‐DL‐leucine
AT: ataxia‐telangiectasia
ATM: ataxia telangiectasia mutated
AT‐NEST: AT neurological examination scale toolkit
eDSP: dexamethasone sodium phosphate encapsulated in erythrocytes (Erydex)
ICARS: international cooperative ataxia rating scale
mICARS: modified international cooperative ataxia rating scale
NAD+: nicotinamide adenine dinucleotide
NALL: N‐Acetyl‐L‐leucine
NEAT: evaluate the neurological effects of EryDex on subjects with AT
NR: nicotinamide riboside
OLE NEAT: open‐label extension of EryDex study
PICOS: population/participants, intervention, comparator/control, outcomes, and study design
RCT: randomized controlled trial
RmICARS: rescored modified International Cooperative Ataxia Rating Scale
RoB 2: revised Cochrane risk‐of‐bias tool
SARA: scale for the assessment and rating of ataxia
SCAFI: spinocerebellar ataxia functional index

## 1 Introduction

Ataxia‐telangiectasia (AT) is a rare autosomal recessive multisystem disorder with prominent progressive neurodegeneration, typically presenting in early childhood [1, 2, 3, 4].

AT is caused by biallelic pathogenic variants in the ATM gene located on chromosome 11q22.3. The ATM gene encodes a serine/threonine protein kinase involved in several cellular processes, including the response to double‐stranded DNA breaks induced by ionizing radiation, oxidative stress, and alkylating agents [5].

The broad biological functions of the ATM protein contribute to the marked clinical heterogeneity and multisystemic involvement of AT. Loss of ATM activity disrupts DNA damage response pathways and cell‐cycle checkpoint control, ultimately resulting in cellular genomic instability. These molecular dysfunctions underlie both the progressive neurological deterioration and the systemic manifestations associated with the disease [1].

Progressive cerebellar atrophy represents the hallmark neurological feature of AT, with cerebellar ataxia typically developing between 2 and 5 years of age in children who had previously achieved normal developmental milestones [1, 6]. In the classic phenotype, loss of independent ambulation usually occurs by approximately 10 years of age, often accompanied by progressive sensorimotor axonal neuropathy [6]. However, disease progression is highly variable, and although genotype–phenotype correlations are increasingly recognized, they remain only partially understood [7].

Beyond cerebellar dysfunction, the neurological phenotype of AT encompasses a wide spectrum of movement disorders, including dystonia, choreoathetosis, myoclonus, and tremor. Parkinsonism may also develop during the second or third decade of life. Among these, dystonia is often one of the most disabling symptoms [8, 9]. Oculomotor apraxia, strabismus, and dysarthria further contribute to the clinical heterogeneity of the disorder.

Residual ATM kinase activity is associated with a milder disease phenotype characterized by later onset, slower progression, and longer survival, with dystonia occasionally predominating over cerebellar ataxia [10, 11].

Despite advances in understanding AT pathophysiology, therapeutic options for neurological symptoms remain largely supportive [12]. Over the past two decades, interventional studies have explored pharmacological strategies targeting molecular pathways involved in AT‐related neurodegeneration, including oxidative damage and mitochondrial dysfunction [13, 14, 15]. However, no systematic review has synthesized this evidence for pediatric patients with AT.

Childhood represents the period of most rapid neurodegenerative progression, with accelerated decline occurring between 6 and 10 years of age. This period may therefore represent the optimal window for therapeutic intervention [13]. This systematic review aims to evaluate the efficacy and safety of pharmacological interventions for neurological symptoms in pediatric patients with AT, assessed through standardized clinical scales.

## 2 Methods

### 2.1 Protocol Registration

This systematic review was conducted following established methodological standards for systematic reviews of interventions, as outlined in the Cochrane Handbook for Systematic Reviews of Interventions [16].

The review protocol was prospectively registered in the International Prospective Register of Systematic Reviews (PROSPERO) on November 6, 2025 (registration number: CRD420251184721). Given the nature of the study, ethics approval was not required. Furthermore, no written consent has been obtained from the patients as there is no patient‐identifiable data included.

### 2.2 Study Identification and Selection

The identification and selection of eligible studies were conducted in three sequential phases. First, a predefined search algorithm was developed and applied to selected scientific databases and registries to identify potentially relevant studies. Second, duplicates were removed and titles and abstracts of retrieved records were screened against predefined eligibility criteria. Third, full‐text articles of potentially eligible studies were assessed for inclusion, and data were extracted from studies meeting all eligibility criteria.

### 2.3 Search Strategy

A comprehensive literature search was conducted across three major electronic databases in health and biomedical sciences: MEDLINE (via PubMed), Scopus, and Web of Science. The search strategy was designed to maximize sensitivity while remaining feasible, following principles outlined in the Cochrane Handbook [16]. Additionally, the reference lists of all included studies were manually examined to identify additional relevant publications, and the U.S. National Institutes of Health clinical trials registry (ClinicalTrials.gov) was searched to identify ongoing or unpublished trials.

All studies published from database inception through December 22, 2025, were considered, without applying publication year restrictions.

The following search algorithm was applied across databases: *(“Ataxia Telangiectasia” OR “Ataxia‐Telangiectasia” OR “Hereditary Ataxia” OR AT OR A‐T) AND (“Treatment” OR “Therapy” OR “Drug” OR “Pharmacological” OR “Medication”) AND (“Safety” OR “Efficacy” OR “Improvement”)*. Minor adaptations to the search syntax were made to accommodate differences in database indexing systems and search functionalities, without altering the conceptual framework of the search strategy. ClinicalTrials.gov was queried using the condition term “Ataxia Telangiectasia” combined with intervention‐related keywords *(“drug” OR “pharmacological”)*.

### 2.4 Eligibility Criteria for Study Inclusion

Records retrieved from electronic databases and other sources were imported into Zotero reference management software (version 6.0) and subsequently transferred to Excel to facilitate duplicate identification and screening. Duplicates were removed manually by two independent investigators. Duplicates were identified based on matching journal title, publication year, author list, volume, issue, and page numbers. All duplicates were documented and reported in the PRISMA flowchart [17].

Following duplicate removal, two independent reviewers screened titles and abstracts of remaining records to identify potentially eligible studies. Prior to full screening, a pilot calibration exercise was conducted on a random subset of 50 records to ensure consistent application of eligibility criteria and adequate inter‐rater reliability. Reviewers were not blinded to each other's assessments. Disagreements were resolved through discussion until consensus was reached; a third reviewer resolved unresolved discrepancies. All screening decisions were documented.

Studies were included if they met the following criteria:

- Population: Children and adolescents aged ≤ 18 years with a confirmed clinical and genetic diagnosis of AT;
- Intervention: Any therapeutic intervention targeting neurological symptoms or aimed at slowing neurological progression, including pharmacological agents, dietary supplements, or rehabilitative approaches;
- Comparator (when available): Placebo, standard symptomatic therapy, usual care, no treatment, historical controls, baseline (pre–post intervention) comparisons, or other active interventions;
- Outcomes: Studies reporting standardized assessments of neurological function using validated clinical scales (e.g., International Cooperative Ataxia Rating Scale (ICARS), Scale for the Assessment and Rating of Ataxia (SARA), or other validated neurological outcome measures);
- Study design: Randomized controlled trials (RCTs), non‐randomized controlled trials, prospective or retrospective cohort studies, longitudinal observational studies, case–control studies, and case series reporting ≥ 3 participants;
- Peer‐reviewed publications in English.

Studies were excluded if they met any of the following criteria:

- Enrolled participants older than 18 years without separate reporting of pediatric data;
- Included participants with other genetically inherited forms of ataxia without separate reporting of AT‐specific data;
- Did not report standardized assessments of neurological function using validated clinical scales;
- Single‐patient case reports;
- Animal or in vitro studies;
- Gray literature, narrative or systematic reviews, editorials, letters, conference abstracts, or other unpublished materials;
- Published in languages other than English;
- Lacked a clearly defined intervention duration or follow‐up period.

Full‐text articles were retrieved for all records that passed title and abstract screening. Three articles were excluded due to unavailability of the full text despite attempts to contact authors and institutional libraries. The reference lists of all included studies were manually examined to identify additional relevant publications through backward citation searching. Any newly identified citations underwent the same full eligibility assessment process. Studies enrolling mixed populations (e.g., both pediatric and adult participants, or AT and other ataxias) were considered eligible only if data specific to pediatric participants with AT were reported separately or could be obtained from the authors [17].

### 2.5 Data Extraction

Data extraction was performed independently by two reviewers using a standardized, piloted data extraction form based on the PICOS framework (Population, Intervention, Comparison, Outcomes, Study design). The following variables were extracted from each included study:

- Study characteristics: Authors, year of publication, country, study design, funding source;
- Population characteristics: Sample size, age range, sex distribution, genetic confirmation of AT diagnosis (including specific ATM variants when reported), baseline disease severity;
- Intervention characteristics: Type of intervention, treatment modality, dose, frequency, duration, and route of administration;
- Comparator characteristics (when applicable): Type of control group, sample size, baseline characteristics;
- Outcome measures: Primary and secondary neurological outcomes, assessment tools and validated clinical scales (e.g., ICARS, SARA), timing of assessments, definition of clinically meaningful change;
- Results: Main findings for neurological outcomes, effect sizes when reported, measures of precision (confidence intervals or standard errors), statistical significance;
- Safety and tolerability: Adverse events, serious adverse events, withdrawals due to adverse events, tolerability assessments;
- Follow‐up: Duration of follow‐up and loss to follow‐up.

To minimize the risk of double‐counting participants and outcomes, all included studies were carefully examined to identify potential sample overlaps based on authorship, study centers, recruitment periods, demographic characteristics, sample sizes, and trial registration numbers. Supplementary data from related publications was extracted when it provided additional relevant information not available in the primary source. In cases of missing, ambiguous, or incomplete data, corresponding authors were contacted via email.

### 2.6 Risk of Bias Assessment

Two reviewers assessed risk of bias independently. For randomized controlled trials, the revised Cochrane Risk of Bias tool for randomized trials (RoB 2) was applied, evaluating bias arising from the randomization process, deviations from intended interventions, missing outcome data, measurement of the outcome, and selection of the reported result [18]. For single‐arm, open‐label interventional studies and pre‐post studies without control groups, the National Heart, Lung, and Blood Institute (NHLBI) Quality Assessment Tool for Before‐After (Pre‐Post) Studies With No Control Group was used [19]. Each domain in RoB 2 was rated as “low risk,” “some concerns,” or “high risk” of bias, with an overall risk of bias judgment derived according to the algorithm specified in the tool. For studies assessed with the NHLBI tool, an overall quality rating of “good,” “fair,” or “poor” was assigned based on the number and severity of methodological limitations identified. Discrepancies between reviewers were resolved through discussion or, when necessary, consultation with a third reviewer.

## 3 Results

### 3.1 Search Results

**FIGURE 1:**
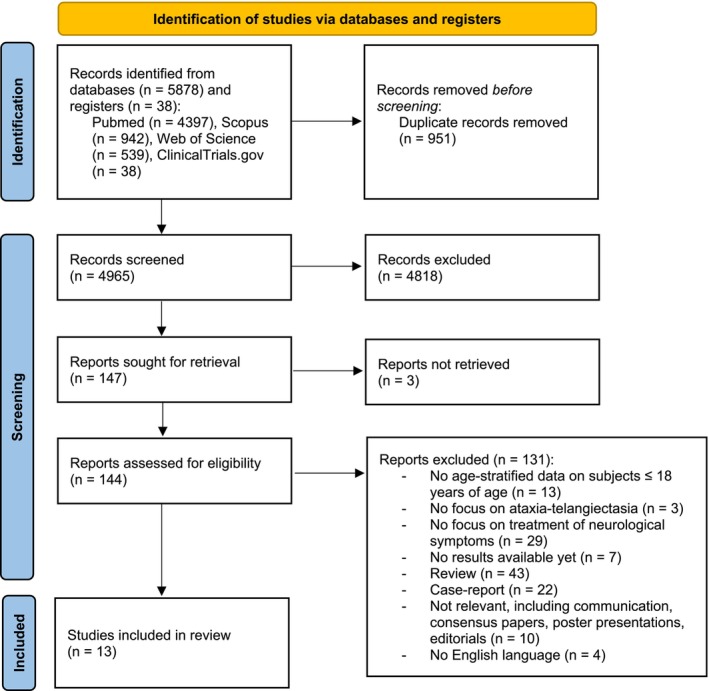
PRISMA 2020 flow diagram for systematic review, including searches of databases and registers.

The systematic literature search across international databases and registries identified a total of 5916 potentially eligible records. After duplicate removal, 4965 unique records underwent title and abstract screening. Of these, 147 publications passed the initial screening phase and were retrieved for full‐text assessment. Three articles were excluded due to unavailability of the full text despite attempts to contact authors. Applying the predefined inclusion and exclusion criteria to the remaining 144 full‐text articles resulted in 13 studies that met all eligibility criteria and were included in this systematic review (Figure 1). Due to the limited number of eligible studies with comparable designs and interventions (*n* = 3 RCTs evaluating different treatments), substantial methodological heterogeneity, and variability in outcome measures and follow‐up durations, meta‐analysis was not feasible. Findings were therefore synthesized narratively.

### 3.2 General Characteristics of Included Studies

All included studies were published between 2008 and 2024. The 13 studies comprised three randomized controlled trials (RCTs) and ten single‐arm, open‐label interventional studies, enrolling a total of 314 participants (median: 10 participants per study). Among participants with available age data (*n* = 298), the weighted mean age was 10.87 years, with all participants aged ≤ 18 years. The overall sex distribution was 55.4% male (165/298) and 44.6% female (133/298).

Studies were conducted across diverse geographical regions: twelve were single‐center studies (six in Italy, one each in Germany, Norway, the Netherlands, Israel, Iran, and Japan), while one was a multicenter multinational RCT. Regarding study design, three studies were RCTs (one with a crossover design) and ten were single‐arm, open‐label interventional studies.

In single‐arm studies, pharmacological interventions included: oral betamethasone (*n* = 4), erythrocyte‐encapsulated dexamethasone sodium phosphate (eDSP; EryDex) (*n* = 2), nicotinamide riboside (NR) (*n* = 2), acetyl‐DL‐leucine (ADLL) (*n* = 1), and amantadine sulfate (*n* = 1). The three RCTs compared the following interventions with placebo: oral betamethasone, eDSP, and N‐acetyl‐L‐leucine (NALL) with a crossover design.

Primary outcomes across studies focused on neurological symptom efficacy, assessed using several neurological rating scales. Follow‐up duration ranged from a minimum of 10 days to a maximum of 48 months (median: 6 months). Secondary outcomes primarily concerned safety and treatment‐related adverse events.

Table 1 summarizes the general characteristics of the included studies.

**TABLE 1:** General characteristics of the included studies, grouped by treatment.

Treatment	Bibliography	Country	Design	Partecipants		Dose	Way	Follow‐up
				*N* < 19 years (% F)	Mean age (± SD)			
Betamethasone	Broccoletti et al. (2008) [20]	Italy	SAT‐OL	4 (50%)	16.3 (NA)	0.1 mg/kg/die for 10 d	Os	10 days
	Broccoletti et al. (2011) [21]	Italy	SAT‐OL	4 (50%)	12.7 (±4.96)	Cycle I: 0.01 mg/kg/die (20 days) Cycle II: 0.03 mg/kg/die (20 days)	Os	80 days
	Zannolli et al. (2012) [22]	Italy	RCT	13 (46%)	9.7 (±2.64)	2 cycles of 0.01 mg/kg/die for 10 days	Os	90 days
	Cirillo et al. (2018) [23]	Italy	SAT‐OL	8 (55%)	10.78 (±5.89)	Cycle I: 0.001 mg/kg/die (30 days) Cycle II: 0.005 mg/kg/die (30 days) Cycle III: 0.01 mg/kg/day (30 days)	Os	8 months
	Hasegawa et al. (2019) [24]	Japan	SAT‐OL	6 (50%)	9.2 (NA)	0.02 mg/kg/day for 24 m	Os	48 months
eDSP	Chessa et al. (2014) [25]	Italy	SAT‐OL	22 (50%)	11.2 (±3.5)	500 mg every 28 days for 6 months	Iv	6 months
	Leuzzi et al. (2015) [26]	Italy	SAT‐OL	4 (0%)	10.6 (±2.8)	500 mg every 28 days for 18 months	Iv	24
	Zielen et al. (2024) [13]	Multicenter Multinational	RCT	175 (48%)	10 (±4.1)	5–10 mg or 14–22 mg based on weight/age every 28 days for 6 months	Iv	12 months
NR	Veenhuis et al. (2021) [14]	Netherlands	SAT‐OL	17 (37%)	17.5 (±15.0)	25 mg/kg/die for 4 months	Os	6 months
	Presterud et al. (2024) [27]	Norway	SAT‐OL	7 (60%)	13.2 (NA)	Starting at 150 mg/die up to 500 mg/die for 18 months	Os	24 months
Acetyl‐DL‐leucine	Brueggemann et al. (2022) [28]	Germany	SAT‐OL	5 (83%)	16.2 (±5.6)	3 g/die for 1 w, 5 g/die for 3 weeks	Os	4 weeks
NALL	Beyraghi‐Tousi et al. (2024) [29]	Iran	RCT‐Cx	16 (50%)	9.8 (±3.5)	4 g/die, or 3 g/die, or 2 g/die based on weight/age for 6 weeks	Os	16 weeks
Amantadine	Nissenkorn et al. (2013) [30]	Israel	SAT‐OL	17 (29%)	11.2 (±3.9)	7 mg/kg/die for 8 weels	Os	8 weeks

Abbreviations: Cx, crossover; eDSP, dexamethasone sodium phosphate encapsulated in erythrocytes; F, females; Iv, intravenous; M, months; NA, not available; NALL, N‐acetyl‐L‐leucine; NR, nicotinamide riboside; Os, oral; RCT, randomized controlled trial; SAT‐OL, single‐arm trial, open‐label.

### 3.3 Extracted Variables

The primary outcomes of the included studies focused on changes in neurological symptoms associated with cerebellar ataxia, systematically assessed using standardized and validated clinical scales. The most frequently employed instruments included the International Cooperative Ataxia Rating Scale (ICARS), the Scale for the Assessment and Rating of Ataxia (SARA), the AT Neurological Examination Scale Toolkit (AT‐NEST), and the Spinocerebellar Ataxia Functional Index (SCAFI). The principal secondary outcome was the assessment of safety and treatment‐related adverse events.

### 3.4 Neurological Efficacy of Pharmacological Treatments

#### 3.4.1 Corticosteroids

Oral betamethasone. Betamethasone was the most frequently investigated intervention among the included studies [20, 21, 22, 23, 24]. Oral betamethasone, administered in short cycles at escalating doses, consistently demonstrated transient but significant neurological improvements, as reflected by reductions in SARA and AT‐NEST scores. The first pilot study [20] evaluated a 10‐day cycle of oral betamethasone at 0.1 mg/kg/day in six patients, documenting improvement in neurological symptoms. A subsequent study by the same group [21] evaluated very‐low‐dose betamethasone in six responsive patients who received two 20‐day cycles at 0.01 and 0.03 mg/kg/day (10% and 30% of the previously used full dosage); SARA scores significantly improved in all patients at the higher dosage, with clinical improvement already evident at the lower dose. Glucocorticoid Induced Leucine Zipper expression was identified as a potential biomarker of clinical response [21]. A multicenter, observer‐blind study [23] evaluated escalating doses (0.001, 0.005, and 0.01 mg/kg/day) in nine patients and found that four of nine patients benefited at 0.005 mg/kg/day, with only one additional responder at the higher dose; the lowest dose (0.001 mg/kg/day) was ineffective.

In a multicenter, double‐blind, randomized, placebo‐controlled crossover trial [22] involving 13 children with AT, betamethasone reduced the ICARS total score by a median of 13 points in the intention‐to‐treat population and 16 points in the per‐protocol population (median percent decreases of ataxia symptoms of 28% and 31%, respectively).

A long‐term study [24] evaluating two years of continuous low‐dose betamethasone (0.02 mg/kg/day) in six patients confirmed initial transient improvement in five of six participants; however, after two years of treatment, improvement persisted in only one participant, while neurological scores deteriorated in four patients.

Neurological improvements regressed after treatment discontinuation across all studies.

Erythrocyte‐encapsulated dexamethasone sodium phosphate (eDSP). eDSP was developed as a novel drug delivery system allowing slow release of dexamethasone over approximately four weeks following monthly intravenous infusions, thereby avoiding the side effects observed with daily steroid use [25].

In an open‐label phase 2 trial [25] involving 22 children with AT (mean age 11.2 ± 3.5 years), treatment with monthly eDSP infusions for six months led to a significant reduction in ICARS score, with a mean reduction of 4 points in the intention‐to‐treat population (*n* = 22; *p* = 0.02) and 5.2 points in the per‐protocol population (*n* = 18; *p* = 0.01). A significant improvement was also observed in Vineland Adaptive Behavior Scales (VABS) scores at 3 and 6 months (*p* < 0.0001) [25]. A large inter‐patient variability in the incorporation of dexamethasone sodium phosphate into erythrocytes was observed, with an evident positive effect of higher infusion dose on ICARS score decline; moreover, a more marked improvement was found in less neurologically impaired patients [25].

A 19‐month extension study involving a subgroup of four patients suggested that eDSP treatment may delay the natural progression of the disease, confirming continuous neurological response and a favorable safety profile [25, 26].

However, in the ATTeST study [13], a multicenter, randomized, double‐blind, placebo‐controlled phase 3 trial conducted at 22 centers in 12 countries, eDSP at both low dose (approximately 5–10 mg) and high dose (approximately 14–22 mg) did not significantly affect mean mICARS scores compared to placebo after 6 months of treatment in the modified intention‐to‐treat population (least squares mean difference −1.37 [95% CI −2.932 to 0.190] for low dose and −1.40 [−2.957 to 0.152; *p* = 0.0765] for high dose). The trial enrolled 176 children aged ≥ 6 years with preserved autonomous gait, of whom 175 received at least one dose of treatment. Notably, a prespecified subgroup analysis revealed that children aged 6–9 years treated with high‐dose eDSP experienced a significant reduction in mean mICARS scores compared to placebo (least squares mean difference −2.79 [95% CI −5.090 to −0.480]; nominal *p* = 0.0185), as well as in ICARS scores (least squares mean difference −4.55 [95% CI −8.478 to −0.628]; nominal *p* = 0.0236). The authors emphasized that treatment of neurodegenerative disorders is often more effective earlier in the disease course before significant neuronal damage occurs, and that future randomized therapeutic trials in patients with AT should be stratified by age and adequately powered within each age group [13].

#### 3.4.2 Nicotinamide Riboside

Daily treatment with nicotinamide riboside (NR), a form of vitamin B3 and NAD+ precursor, for four months resulted in improvements in SARA and AT‐NEST scores in an open‐label proof‐of‐concept study involving 24 patients with AT (mean age 12.5 years, range 6–25 years) [14]. Specifically, SARA scores improved by a mean of 1.1 points (95% CI 0.4–1.9; *p* = 0.006) and AT‐NEST scores improved by 2.9 points (95% CI 0.6–5.2; *p* = 0.02) [14]. NR treatment was also associated with increased serum immunoglobulin G concentrations in immunodeficient patients [14].

These findings were confirmed in a subsequent single‐arm study [27] evaluating 18 months of NR treatment in 25 patients (mean age 14.9 years, range 6–34 years). Improvements were observed primarily in coordination subscores and eye movements, with SARA scores improving by a mean of 1.3 points (95% CI 0.3–2.3; *p* = 0.02) and AT‐NEST scores by 3.9 points (95% CI 1.9–5.9; *p* < 0.001) [27].

In both studies, neurological status worsened upon treatment discontinuation, with scores returning to baseline levels or worse. ICARS scores showed no significant pre‐ vs. post‐treatment differences with NR [14, 27].

#### 3.4.3 Leucine Derivatives (Acetyl‐DL‐Leucine and N‐Acetyl‐L‐Leucine)

Acetyl‐DL‐leucine (ADLL) and N‐acetyl‐L‐leucine (NALL) are modified amino acids that have been investigated for their potential symptomatic effects on cerebellar ataxia.

In an open‐label study [28], six patients with AT (age range 6–18 years) were treated with ADLL at 3 g/day for one week followed by 5 g/day for three weeks to one year. Treatment resulted in a significant reduction of the mean total SARA score from 22.1 (SD 5.88) at baseline to 18.0 (SD 5.39) after one month of therapy (*p* = 0.0028). Additionally, all patients showed gaze‐holding deficits, and three patients had central‐position downbeat nystagmus; mean slow‐phase velocity of this nystagmus improved from 5.57°/s to 4.7°/s after one month on treatment (*p* = 0.046). However, SCAFI scores demonstrated poor sensitivity to change in this population [28].

In contrast, a randomized, double‐blind, placebo‐controlled, crossover clinical trial [29] evaluating NALL (the more pharmacologically active enantiomer) in 16 patients with AT (mean age 9.8 ± 3.5 years) showed no significant differences compared to placebo after six weeks of treatment. NALL treatment had no significant effects on SARA scores, SCAFI‐9HPT (9‐hole peg test) for either hand, or SCAFI‐8MWT (8‐m walking time). Physical Health scores in both child self‐report and parent proxy‐report did not significantly change in the treatment group compared to placebo [29].

#### 3.4.4 Amantadine

Amantadine, an NMDA receptor antagonist with dopaminergic properties, was evaluated in a single open‐label study [30] involving 17 patients with AT (mean age 12.6 ± 6.2 years, range 3–25 years). Treatment with amantadine at 5 mg/kg/day (maximum 200 mg/day) for eight weeks resulted in a 29% mean improvement in the composite AT score, with a reduction from 76.47 at baseline to 54.94 post‐treatment [30]. Improvements were observed across multiple domains, including gait, stance, upper limb coordination, and speech [30].

Table 2 provides a summary of the results from the studies included.

**TABLE 2:** Main neurological outcomes and adverse events for each treatment evaluated in the included studies.

Treatment	Bibliography	Scale	Baseline (mean, SD)	Post‐treatment	Outcome (Δ mean)	Adverse events
Betamethasone	Broccoletti et al. (2008) [20]	SARA	24 (±6.29)	20.0 (±8.15)	−4.0	Lymphocyte count increase
	Broccoletti et al. (2011) [21]	SARA	23 (± 5.6)	17.5 (±6.5)	−5.5*	Mild to moderate GI
	Zannolli et al. (2012) [22]	ICARS	45.46 (±13.26)	Not reported	−13.0* (95% CI: −19, −5.5)	Mild treatment‐related: mood swings, weight gain, moon face, asthenia
	Cirillo et al. (2018) [23]	SARA	Not reported	Not reported	Partial reduction in the 55.5% of partecipants	Mild treatment‐related: upper respiratory tract infections/otitis, hypercholesterolemia, hypertension
	Hasegawa et al. (2019) [24]	SARA AT‐NEST	SARA: 18.8 AT‐NEST: 63	SARA: 17 AT‐NEST: 67.2	Δ SARA −1.8* Δ AT‐NEST +4.2	Moderate treatment‐related: secondary adrenal insufficiency, ocular hypertension
eDSP	Chessa et al. (2014) [25]	ICARS	50.6 (±12.8)	46.7 (±10)	−3.9*	Moderate‐to‐severe treatment‐related: recurrent upper respiratory tract infections, bronchopneumonia, hypercholesterolemia, CD4+ lymphocytopenia, hypoferritinemia
	Leuzzi et al. (2015) [26]	ICARS	Continuing treatment group: 52.2 (±7.1) Discontinuation group: 43.0 (±2.9)	Not reported	Treatment group +8.2 versus discontinuation group +17.8**	Not reported
	Zielen et al. (2024) [13]	mICARS	Total sample: 27.9 (±7.0) Low‐dose group: 28.0 (±7.38) High‐dose group: 27.5 (±7.29) Placebo group: 28.4 (±6.53)	Not reported	6–9 year subgroups: high‐dose versus placebo −2.79*	Mild: potentially steroid‐related and irritability; severe: positive sterility test (asymptomatic) and asymptomatic anemia.
NR	Veenhuis et al. (2021) [14]	SARA ICARS	SARA: 21.3 (±9.2) ICARS: 58.3 (± 9.2)	Not reported	Δ SARA −2.4* Δ ICARS −10.1*	Not reported
	Presterud et al. (2024) [27]	SARA ICARS AT‐NEST	SARA: 22.6 (±3.4) ICARS: 62.8 (±10.8) A‐T NEST: 52.7 (±9.7)	SARA: 19.8 (±3.8) ICARS: 54.4 (±15.2) A‐T NEST: 59.1 (±7.9)	Δ SARA −2.8* Δ ICARS −8.4* Δ AT‐NEST +6.4*	Mild GI
Acetyl‐DL‐leucine	Brueggemann et al. (2022) [28]	SARA	22.1 (±5.88)	18.0 (±5.39)	−4.1**	Not reported
NALL	Beyraghi‐Tousi et al. (2024) [29]	SARA SCAFI	SARA pooled: 16.1 (±10.3) SCAFI: not reported	SARA pooled: 10.0 (± 9.3) SCAFI: not reported	SARA and SCAFI: no significant treatment effects	Mild to moderate GI
Amantadine	Nissenkorn et al. (2013) [30]	AT‐score	76.7 (±17.48)	54.94 (±17.5)	−21.8**	Mild to moderate GI

*Note:* Outcomes expressed as difference (Δ) mean ± standard deviation.

Abbreviations: AT‐NEST, Ataxia‐Telangiectasia Neurological Examination Scale Toolkit; eDSP, dexamethasone sodium phosphate encapsulated in erythrocytes; GI, gastrointestinal; ICARS, International Cooperative Ataxia Rating Scale; mICARS, modified International Cooperative Ataxia Rating Scale; NALL, N‐acetyl‐L‐leucine; NR, nicotinamide riboside; SARA, Scale for the Assessment and Rating of Ataxia; SCAFI: Spinocerebellar Ataxia Functional.

* *p* < 0.05.

** *p* < 0.01.

### 3.5 Adverse Events

Oral betamethasone induced dose‐dependent side effects, ranging from mild to severe, including increased susceptibility to upper respiratory tract infections and otitis, metabolic disturbances (weight gain, elevated cholesterol and triglyceride levels), and ocular or systemic hypertension [20, 21, 22, 23, 24].

These adverse effects were more pronounced at higher doses and with prolonged treatment, representing a significant limitation to long‐term steroid use in this population [20, 21, 22, 23, 24].

During eDSP treatment, steroid‐related side effects and irritability were reported in most participants; however, the erythrocyte‐encapsulated delivery system was designed to minimize systemic steroid exposure and associated toxicity [13, 25, 26]. In the ATTeST trial, treatment‐emergent adverse events occurred in 91% of participants in the low‐dose group, 88% in the high‐dose group, and 86% in the placebo group, with most events being mild to moderate in severity [13].

In subjects treated with NR, ADLL, NALL, and amantadine, adverse events were predominantly mild‐to‐moderate gastrointestinal symptoms, including nausea, abdominal discomfort, and transient diarrhea [14, 27, 28, 29, 30].

Treatment discontinuation rates were low across studies (median 0–1 participant per study). Higher discontinuation rates were observed in eDSP studies with extended follow‐up, mainly due to consent withdrawal or early treatment cessation related to the COVID‐19 pandemic, with minimal association with adverse events [13].

A summary of adverse effects for each treatment is presented in Table 2.

### 3.6 Quality Assessment

The risk of bias was assessed using validated tools appropriate to each study design. For the 10 single‐arm open‐label interventional studies, the National Institutes of Health Quality Assessment Tool for Before–After (Pre–Post) Studies With No Control Group was applied. For the three RCTs, the revised Cochrane risk‐of‐bias tool (RoB 2) was used, which evaluates five domains: randomization process, deviations from intended interventions, missing outcome data, measurement of outcomes, and selection of reported results [18, 19]. Among single‐arm studies, 2 of 10 were rated as poor quality, while the remaining 8 were considered of moderate quality. Common methodological limitations included small sample sizes, absence of control groups, lack of blinding, and short follow‐up duration.

Regarding the RCTs, one study was judged to have a low risk of bias, and the other two were classified as having “some concerns.” The primary sources of concern in these trials related to potential deviations from intended interventions and incomplete reporting of randomization procedures. Table 3 provides an overview of the main limitations, the quality assessment process, and the risk‐of‐bias evaluation for each study included.

**TABLE 3:** Quality assessment, risk of bias and main limitations of the included studies, grouped by design and treatment.

Design	Treatment	Bibliography	Quality^a^	Risk of bias^b^	Limitations
SAT‐OL	Betamethasone	Broccoletti et al. (2008) [20]	Moderate		Short treatment duration
		Broccoletti et al. (2011) [21]	Moderate		Selection bias; placebo effect
		Cirillo et al. (2018) [23]	Moderate		Exclusion of partecipants from final analysis
		Hasegawa et al. (2019) [24]	Moderate		Early discontinuation included in final analysis; arbitrary therapeutic modifications due to adverse events
	eDSP	Chessa et al. (2014) [25]	Moderate		Variability in dexamethasone dose; incomplete treatments included in the final analysis
		Leuzzi et al. (2015) [26]	Moderate		Post hoc empirical observation
	NR	Veenhuis et al. (2021) [14]	Low		Possible placebo effect; exclusion of some partecipants in the final analysis
		Presterud et al. (2024) [27]	Moderate		Short duration; incomplete assessments
	Acetyl‐DL‐leucine	Brueggemann et al. (2022) [28]	Low		Limited duration and incomplete assessment in some cases
	Amantadine	Nissenkorn et al. (2013) [30]	Moderate		AT‐score not validated; effect sizes not reported; exclusion of some partecipants from the final analysis
RCT	Betamethasone	Zannolli et al. (2012) [22]		Some concerns	Short duration with variable dosing; possible carry‐over effect
	NALL	Beyraghi‐Tousi et al. (2024) [29]		Some concerns	Limited duration; potential carry‐over effect
	eDSP	Zielen et al. (2024) [13]		Low	Not validated mICARS; treatment delays/omissions; dose variability; per‐protocol analysis

Abbreviations: eDSP, dexamethasone sodium phosphate encapsulated in erythrocytes; NALL, N‐acetyl‐L‐leucine; NR, nicotinamide riboside; RCT, randomized controlled trial; SAT‐OL, single‐arm trial, open‐label.

^a^ Assessed with the NIH Quality Assessment Tool for Before–After (Pre–Post) Studies With No Control Group for single‐arm, open‐label interventional studies.

^b^ Assessed with the revised Cochrane risk‐of‐bias tool (RoB 2) for randomized controlled trials.

## 4 Discussion

This systematic review provides an updated synthesis of the efficacy and safety of pharmacological treatments for cerebellar ataxia in pediatric patients with AT. All included studies were published in the last two decades, reflecting growing interest in targeted therapeutic strategies for rare neurological diseases in pediatrics. However, the evidence base is constrained by the predominance of small, single‐arm, open‐label studies with substantial methodological heterogeneity, which limits the strength and generalizability of conclusions. The following discussion integrates the interpretation of findings with a critical appraisal of the methodological limitations that affect the certainty of the available evidence.

Of the 13 studies analyzed, only three were RCTs, while the remaining ten were single‐arm open‐label interventional studies. According to the GRADE framework, non‐randomized studies of interventions are initially rated as low‐certainty evidence due to the absence of randomization and control groups, which introduces substantial risk of confounding, selection bias, and performance bias [31].

Single‐arm trials present particular challenges for estimating comparative effectiveness, including threats to validity, reliability, and statistical power, as well as the inability to distinguish treatment effects from natural disease fluctuation, regression to the mean, or placebo response [32]. Our risk of bias assessment confirmed these concerns: 8 of 10 single‐arm studies were rated as moderate quality ratings and 2 as poor quality using the National Institutes of Health Quality Assessment Tool, while among the three RCTs, only one was judged to have low risk of bias. Common methodological limitations across studies included small sample sizes (median of 10 participants per study; total *n* = 314), absence of blinding, heterogeneous outcome measures, and variable follow‐up durations ranging from 10 days to 48 months. These limitations are consistent with broader challenges in rare disease research, where small, geographically dispersed patient populations and underlying clinical heterogeneity make robust study designs difficult to implement [33, 34]. Notably, the three RCTs, representing the highest‐quality evidence, consistently showed more modest or null effects compared to the more optimistic results from open‐label studies, a pattern consistent with the broader evidence showing that trials at high or unclear risk of bias tend to produce larger treatment effect estimates. This discrepancy underscores the importance of interpreting open‐label findings with caution and highlights the need for adequately powered, controlled trials to establish treatment efficacy in AT.

Regarding specific interventions, glucocorticoids, including oral betamethasone and eDSP, have been the most extensively investigated. Betamethasone demonstrated transient clinical benefits on neurological symptoms across multiple studies; however, these improvements consistently regressed upon treatment discontinuation, and significant dose‐dependent systemic toxicity limits the feasibility of long‐term administration [20, 21, 22, 23, 24]. Critically, the absence of placebo‐controlled data in most betamethasone studies precludes definitive conclusions about efficacy beyond natural fluctuation in disease course or placebo effects, a limitation that is particularly relevant in AT given its non‐linear progression characterized by periods of rapid neurological deterioration alternating with stabilization.

In an attempt to improve glucocorticoid safety and tolerability, eDSP was developed as an alternative delivery approach aimed at enabling long‐term treatment while minimizing systemic toxicity. Early‐phase studies suggested preliminary signals of potential benefit, particularly in patients with less severe neurological impairment at baseline and in those with optimized pharmacological erythrocyte loading. However, these results were derived from small, uncontrolled studies and must be interpreted cautiously [25, 26]. The phase 3 ATTeST trial, despite being the largest and most rigorous study to date, did not demonstrate overall efficacy in the modified intention‐to‐treat population, although a prespecified subgroup analysis suggested potential benefit in children aged 6–9 years receiving high‐dose eDSP [13]. This finding aligns with the hypothesis of a “window of sensitivity” in early disease stages but requires confirmation in adequately powered, age‐stratified trials [13]. Notably, the subsequent NEAT phase 3 trial (NCT06193200) also failed to meet its primary endpoint (mean RmICARS difference −1.30, *p* = 0.0851), leading to discontinuation of the open‐label extension program (NCT06664853) [35].

The failure of the NEAT trial, despite its rigorous double‐blind, placebo‐controlled design, raises important questions about the applicability of conventional RCT methodology to rare neurodegenerative diseases with heterogeneous progression. Several design‐related factors may have contributed to the negative outcome. First, the assessment interval of 21–30 days between doses may have been suboptimal for capturing transient neurological improvements; earlier open‐label studies employed more frequent assessments (weekly or biweekly) that may have been more sensitive to short‐term fluctuations in neurological status. Second, the substantial inter‐patient variability in dexamethasone loading into autologous erythrocytes, a well‐documented phenomenon in phase 2 studies, may have introduced pharmacokinetic heterogeneity that diluted treatment effects in the intention‐to‐treat analysis. Third, the non‐linear progression of AT, characterized by periods of rapid deterioration alternating with stabilization, poses fundamental challenges for parallel‐group designs with fixed endpoints.

Recent simulation studies comparing trial designs in autosomal recessive cerebellar ataxias have demonstrated that longitudinal modeling approaches using non‐linear mixed‐effect models achieve substantially higher power (88%) compared to standard end‐of‐treatment analyses (36%) in detecting disease‐modifying effects [36]. Furthermore, delayed‐start designs may offer advantages in rare neurodegenerative diseases by allowing all patients to receive treatment while maintaining the ability to detect treatment effects [34, 36]. These methodological considerations suggest that the failure of eDSP in phase 3 trials may reflect limitations of conventional RCT design in this population rather than definitive evidence of inefficacy, particularly given the consistent signals of benefit observed in younger patients across multiple studies. Preclinical and clinical studies have also shown that eDSP can modulate ATM gene splicing, resulting in the production of transcript variants known as mini‐ATM or ATMdexa1, which include the kinase domain essential for protein function, suggesting that eDSP may also exert molecular effects on DNA repair and genomic stability [37, 38]. In younger patients, a correlation has been observed between mini‐ATM levels and improvements in ICARS total score, supporting the hypothesis that induction of these variants may contribute to slowing neurodegenerative progression; however, these mechanistic observations remain preliminary and require validation in larger, controlled studies [38]. The tolerability profile of eDSP appeared favorable across studies, with 99% of long‐term treated participants in the ATTeST extension experiencing only mild adverse events, in the absence of adverse effects typically associated with long‐term glucocorticoid use.

Beyond glucocorticoids, alternative therapeutic approaches targeting key molecular pathways in AT have emerged. NR, targeting NAD^+^ depletion and cellular energy metabolism [14, 27], showed improvements in SARA and AT‐NEST scores in two open‐label studies [14, 27]. However, the absence of control groups and the regression of benefits upon treatment discontinuation limit causal inference. The discordance between SARA/AT‐NEST improvements and unchanged ICARS scores raises questions about outcome measure sensitivity and the consistency of treatment effects across different assessment tools: a concern that extends to the broader evidence base in this review. Without placebo‐controlled data, it remains unclear whether the observed improvements reflect true pharmacological effects or natural disease fluctuation, regression to the mean, or placebo response. An ongoing randomized placebo‐controlled trial (NCT06324877) is currently evaluating NR efficacy in AT and may provide the controlled data necessary to establish whether the observed benefits reflect true pharmacological effects.

Similarly, leucine derivatives (ADLL and NALL) have yielded mixed results. A single‐arm study of ADLL demonstrated SARA score reductions after four weeks [28]. For NALL, a small RCT in 16 pediatric AT patients showed no significant benefit on SARA or SCAFI scores after six weeks compared to placebo [29]. Preliminary findings from phase 2 IB1001‐203 study (NCT03759678), presented at MDS 2025, indicated that one year of NALL treatment in 12 AT patients resulted in a mean SARA score reduction of 2.25 points from baseline. More recently, IntraBio announced positive results from the pivotal phase 3 IB1001‐301 trial of NALL for AT in January 2026, reporting that the trial met its primary endpoint with statistically significant improvement in neurological function compared to placebo. NALL has already received FDA approval for neurological manifestations of Niemann‐Pick disease type C [39]. If confirmed in peer‐reviewed publication, these findings would represent a potential breakthrough; however, these unpublished data should be interpreted with caution pending full disclosure of methodology, effect sizes, and safety data.

Beyond efficacy considerations, the long‐term safety profile of leucine derivatives in AT patients warrants careful evaluation. Leucine is a potent activator of the mechanistic target of rapamycin complex 1 (mTORC1) signaling pathway, acting through multiple mechanisms [40]. This is particularly relevant in AT, where patients already exhibit a markedly elevated cancer risk due to defective ATM‐mediated tumor suppression. Although the extent to which therapeutic leucine derivatives activate mTORC1 in vivo remains to be determined, and the enantiomeric form (L‐ vs. D‐leucine) may influence this effect, the theoretical concern of exacerbating mTORC1‐driven oncogenic signaling in an already cancer‐prone population merits systematic investigation in long‐term safety studies. Future trials of leucine derivatives in AT should incorporate cancer surveillance protocols and consider monitoring mTORC1 pathway biomarkers.

Regarding other interventions, amantadine, evaluated in a single open‐label study, showed a 29% improvement in composite AT scores after eight weeks of treatment [30]. However, the composite AT score, aggregating ICARS, the Abnormal Involuntary Movement Scale, and the Unified Parkinson's Disease Rating Scale, may primarily reflect effects on parkinsonian symptoms rather than direct improvement in cerebellar ataxia, and the absence of a control group, small sample size (*n* = 17), and short follow‐up duration severely limit the interpretability of these findings [30].

Notably, this systematic review did not identify studies evaluating triheptanoin, an anaplerotic odd‐chain triglyceride that addresses mitochondrial dysfunction through replenishment of tricarboxylic acid cycle intermediates. A recently published phase 2a/b randomized placebo‐controlled dose‐escalation trial by Lynch et al. [41] enrolled 31 participants with AT aged 4 to 37 years (median 16 years) and demonstrated significant improvements in the primary outcome of nasal epithelial cell death (mean difference −9.7%, 95% CI −16.0 to −4.6) as well as in SARA kinetic function subscale (mean difference −5.8, 95% CI −10.4 to −1.2), ICARS gait (mean difference −0.5, 95% CI −0.9 to −0.1) and fine motor disturbance subscales (mean difference −2.7, 95% CI −4.3 to −1.1), and speech intelligibility (mean difference −12.8, 95% CI −21.2 to −4.3). Adverse events included gastrointestinal symptoms (abdominal pain, nausea, vomiting, diarrhea) requiring dose capping at 20% of caloric intake in 38% of participants [41]. This trial represents an important addition to the evidence base, as it is the first adequately powered, placebo‐controlled study to demonstrate efficacy of a metabolic intervention targeting mitochondrial dysfunction in AT. This trial was not included in our systematic review because it did not report separate efficacy or safety outcomes for pediatric participants specifically.

The heterogeneity of outcome measures across studies represents a significant barrier to evidence synthesis. Most quantitative clinical ataxia scales employed in the included studies were validated in populations with slower disease progression than AT [42]. ICARS, for example, was primarily validated in adults and adolescents with focal cerebellar lesions or spinocerebellar ataxias, and its application in pediatric populations may underestimate disease severity, especially in patients who have lost independent ambulation [42, 43, 44, 45]. The mICARS employed in the ATTeST study, while reducing administration time, omits the oculomotor domain and several kinetic items and remains under validation despite regulatory acceptance [13]. Similarly, SARA was originally validated for Friedreich ataxia and may have limited validity in AT [42, 46], while AT‐NEST, despite demonstrating good reliability in adults with AT, is limited by complexity and administration duration that may reduce feasibility in pediatric clinical trials [27, 47, 48]. The discordance between SARA/AT‐NEST improvements and unchanged ICARS scores observed in NR studies exemplifies how outcome measure selection may influence conclusions about treatment efficacy.

Publication bias may also have influenced the available evidence, favoring studies reporting positive or transient results and underrepresenting null findings. The incomplete publication of secondary follow‐up data from several early‐phase studies further limits the ability to assess long‐term efficacy and durability of treatment responses.

Future trials should prioritize age stratification to capture the potential “window of sensitivity” in younger patients, incorporate objective biomarkers alongside clinical scales, and consider adaptive or delayed‐start designs that may be better suited to rare neurodegenerative diseases with heterogeneous progression [34]. Innovative therapeutic approaches including gene therapy and antisense oligonucleotides offer potential for correcting ATM gene defects, although targeted delivery to the central nervous system and long‐term sustainability remain significant challenges [49]. Emerging strategies addressing AT as an oxidative stress disorder, such as triheptanoin, represent promising avenues targeting mitochondrial dysfunction [49]. Given ATM's multifaceted role in DNA repair, cell cycle regulation, oxidative stress response, and mitochondrial function, an integrated therapeutic approach combining symptomatic, metabolic, and potentially gene‐corrective strategies may ultimately prove necessary to address the complex pathophysiology of AT.

## 5 Conclusions

This systematic review indicates that the current evidence supporting pharmacological treatment of cerebellar ataxia in pediatric AT remains insufficient to inform clinical practice. No intervention has demonstrated sustained efficacy in adequately powered controlled trials. The recurring pattern of promising findings from open‐label studies followed by negative phase 3 results, as observed with eDSP and leucine derivatives, highlights a translational gap that warrants methodological innovation, including age‐stratified study designs, objective biomarkers, and analytical approaches that account for non‐linear disease progression. Pending more robust evidence, treatment decisions should be individualized, balancing potential short‐term benefits against toxicity and theoretical risks in this vulnerable population.

## Author Contributions

**Serena Galosi:** writing – review and editing. **Giorgia Urciuolo:** investigation, validation. **Roberto Paparella:** conceptualization, investigation, writing – original draft, methodology. **Fabiola Panvino:** conceptualization, investigation, methodology, writing – original draft. **Francesco Pisani:** supervision. **Carlotta Spagnoli:** writing – review and editing. **Vincenzo Leuzzi:** supervision.

## Funding

The authors have nothing to report.

## Conflicts of Interest

The authors declare no conflicts of interest.

## Data Availability

This study used data from previously published articles that are publicly available. All data analyzed during this study are referenced in the manuscript with full citations.
